# Characteristics of Mesenchymal Stem Cells Are Independent of Bone Marrow Storage Temperatures

**DOI:** 10.1155/2021/6864988

**Published:** 2021-10-19

**Authors:** Valentin Schrodi, Claudia Neunaber, Katrin Bundkirchen, Weikang Ye, Zhida Jiang, Maximilian Petri, Christian Krettek, Sandra Noack

**Affiliations:** ^1^Trauma Department, Hannover Medical School, 30625 Hannover, Germany; ^2^Department of Urology and Urooncology, Clinic of Braunschweig, 38126 Braunschweig, Germany

## Abstract

Mesenchymal stem cells play an important role in regenerative medicine due to their capability of self-renewal and multipotent differentiation. For research or clinical application, bone marrow aspirates are harvested during elective surgeries to isolate MSCs. If an immediate purification of the MSCs is not possible, the bone marrow must be stored. Therefore, the aim of this study was to investigate possible differences of stem cell characteristics regarding the self-renewal capability, the adipogenic, chondrogenic, and osteogenic differentiation, and the expression of surface antigens after different storage conditions of the bone marrow aspirates. Three groups were analysed: the first group was purified immediately after harvesting, the other two groups were processed after they were stored 18 to 24 hours at 22°C (room temperature) or at 4°C. Comparisons between the groups were performed using the Kruskal-Wallis test for nonparametric data. The final results showed no significant difference between the different storage conditions. Therefore, storage of bone marrow aspirates for 18 to 24 hours at room temperature or 4°C is possible without loss of stem cell characteristics.

## 1. Introduction

Mesenchymal stem cells (MSCs) are multipotent self-renewable cells with the capability to differentiate into various cell types like adipocytes, chondrocytes, and osteoblasts [1]. They were primary isolated from bone marrow, but similar populations have also been detected in other human tissues like the dermis, adipose tissue, umbilical cord blood, and placenta [2–4].

Furthermore, MSCs are assumed to have immune modulatory functions as seen during osteoarthritis [5] by influencing the adaptive and innate immune system [6, 7]. These several characteristics predestine MSCs to replace impaired cells or stimulate endogenous repair mechanisms [8]. The capability of mesenchymal stem cells to adhere and proliferate on cell culture surfaces allows them to expand *in vitro* [9]. Therefore, MSCs play a major role in regenerative medicine [10, 11]. In the field of trauma and orthopaedic surgery, MSC-based concepts—for example, seeding of MSCs onto a scaffold [12]—are used as alternative methods to avoid possible complications of the current gold standard to treat large bone defects with autologous or allogeneous bone transplants [13, 14]. However, MSCs are also used in many other clinical disciplines for instance by using the immunoregulatory effect to treat the graft-versus-host disease [15].

In order to develop further applications of stem cells for the clinical practice, research with MSCs is indispensable. In our department, the isolation of MSCs usually occurs immediately after the harvesting of bone marrow aspirates. At night or at weekends, a direct processing of the bone marrow is not possible due to staff shortages. In this case, the bone marrow aspirate has to be stored overnight. Therefore, the aim of our study was to investigate the influence of a longer storage (18 to 24 hours) at different temperatures on the characteristics of human bone marrow-derived stem cells (hBMSCs) regarding the self-renewal capability, the adipogenic, chondrogenic, and osteogenic differentiation potential, and the expression of surface proteins.

## 2. Materials and Methods

### 2.1. Ethical Approval and Purification of hBMSCs

For studies involving human tissues, ethical approval of the study protocol and the process of purifying and analysing the hBMSCs was obtained by the ethical committee of the Hannover Medical School (Ethic No. 2562). Furthermore, a voluntary written informed consent was obtained from each donor, and all personal data were anonymised. A total of six donors were examined.

Human bone marrow was harvested by iliac crest aspiration during elective standard orthopaedic or trauma surgery at the Trauma Department of the Hannover Medical School. After immediate transfer of the bone marrow aspirate to the laboratory, the bone marrow was divided into three groups. The first group was purified immediately (group 0 h), the other two groups were processed after they were stored 18 to 24 hours at room temperature (RT; 22°C) or at 4°C. The syringe in which the bone marrow was harvested was used for storage.

Afterwards, the hBMSCs were isolated by density gradient centrifugation for 30 minutes at 500 × g without brake using a synthetic polysaccharide-epichlorohydrin-copolymer (Biocoll®, Biochrom, Berlin, Germany) as described before [16]. Shortly, the interphase which contains the mononuclear cells was extracted, washed, and seeded in MSC growth medium (DMEM FG 0415 (Biochrom)) with 10% (*v*/*v*) Fetal Bovine Serum (HyClone®FBS, Fisher Scientific, Schwerte, Germany), 20 mM 4-(2-hydroxyethyl)-1-piperazineethanesulfonic acid (HEPES, Biochrom), 1% (100 U/ml/100 *μ*g/ml) penicillin/streptomycin (Biochrom), and 2 ng/ml human recombinant fibroblast growth factor 2 (FGF2, PeproTech, Hamburg, Germany). The cells were now in passage 0 (P0). Cells were cultured at 37°C and 5% CO_2_ until a density of 70–80% was reached [16]. Passaging was performed with 0.025% Trypsin-EDTA solution (Biochrom), and cells were seeded at a density of 2 × 10^3^ cells per cm^2^ for the next passage. All experiments were performed in passage 4 (P4) or as otherwise stated.

### 2.2. Cell Amount and Colony Forming Unit-Fibroblast Assay (CFU-F Assay)

The total number of cells in 1 ml of bone marrow was determined in P0 by counting the cells using a Neubauer counting chamber. To analyse the self-renewal potential of the hBMSCs, two CFU-assays were conducted in P1 and P4. Double determination of the cells was performed in six-well plates (Greiner Bio-One GmbH, Frickenhausen, Germany) in decreasing concentrations of 500, 250, and 125 cells per well. CFUs were cultivated for 10 days at 37°C and 5% CO_2_. Afterwards, cells were fixed with methanol (Merck, Darmstadt, Germany) and dyed for 30 minutes with 1% crystal violet solution (Merck). The colony number was counted to determine the percentage of clones per 100 seeded cells.

### 2.3. Flow Cytometry (FC)

For flow cytometry 1 × 10^7^ cells were used. In P4, cells were detached using 0.025% trypsin-EDTA solution and washed twice with FC buffer (2% (*v*/*v*) FBS in PBS). The centrifugation steps were performed at 400 × g and 4°C for two minutes. Afterwards, the cells were incubated with appropriate fluorochrome-conjugated antibodies (Biolegend, Koblenz, Germany; Table 1) for 60 minutes at 4°C in the dark. This process was followed by two washing steps with FC buffer. The cells were analysed on a FACS Canto (BD Biosciences, Heidelberg, Germany) recording 3 × 10^4^ cells as described before [17]. Dead cells were excluded by using scatter parameters using the BD FACS Diva Software and Flowing Software version 2.5.0.

### 2.4. *In Vitro* Differentiation and Histological Staining

The adipogenic, chondrogenic, and osteogenic differentiation potential of stem cells was examined by inducing the process with convenient differentiation media. Each induced cell group had a concomitant control group. All stem cells were stored in a cell culture incubator at 37°C and 5% CO_2_.

#### 2.4.1. Adipogenic *In Vitro* Differentiation

For adipogenic differentiation, hBMSCs were seeded in six-well plates containing 150,000 cells per well. After 24 hours, the differentiation process was initiated by adipogenic medium consisting of DMEM FG0435 (500 ml (*v*/*v*), Biochrom), dexamethasone (1 *μ*M (*v*/*v*), Sigma Aldrich), indomethacin (60 *μ*M (*v*/*v*), Sigma Aldrich), 3-isobutyl-1-methylxanthine (IBMX, 500 *μ*M (*v*/*v*), Sigma Aldrich), insulin (10 *μ*g/ml (*v*/*v*), Sigma Aldrich), 4-(2-hydroxyethyl)-1-piperazineethanesulfonic acid (HEPES, 20 mM (*v*/*v*), Biochrom), penicillin/streptomycin (1% (*v*/*v*), Biochrom), and Fetal Bovine Serum (FBS, 20% (*v*/*v*), Hyclone®). The control medium consists of DMEM FG0415 (500 ml (*v*/*v*)), 4-(2-hydroxyethyl)-1-piperazineethanesulfonic acid (HEPES, 20 mM (*v*/*v*)), Fetal Bovine Serum (FBS, 10% (*v*/*v*), Hyclone®), and penicillin/streptomycin (1% (*v*/*v*)) and was also used for the control group of the osteogenic differentiation. Media were replaced every 7 days. Cells for histological analysis were fixed in 4% formalin solution on days 0, 7, 14, 21, and 28 and stained with Oil Red O (5 g/l in 60% Isopropanol, Roth) for 25 minutes. Photographs of each well with a 100x magnification were taken on the microscope CKX41 (Olympus, Tokyo, Japan). For quantitative analysis, the degree of adipogenic differentiation was assigned by evaluating the average of the stained area related to the total area. Therefore, a self-written image processing tool that referred to the OpenCV library (version 4.1.0) was used. Three representative images were assessed to ensure valid results. Based on different hue and saturation values, the stained area was kept apart from the background. The threshold levels were set manually by evaluating the specific parameters using a representative image. This data was used for the whole examination to facilitate a consistent assessment.

#### 2.4.2. Osteogenic *In Vitro* Differentiation

Except the differentiation medium and the days of fixation, the osteogenic differentiation procedure was identical to the adipogenic differentiation described above. The differentiation medium for osteogenesis consisted of DMEM FG0415 (500 ml (*v*/*v*)), 4-(2-hydroxyethyl)-1-piperazineethanesulfonic acid (HEPES, 20 mM (*v*/*v*)), Fetal Bovine Serum (FBS, 10% (*v*/*v*), Hyclone®), penicillin/streptomycin (1% (*v*/*v*)), dexamethasone (100 nM (*v*/*v*)), ascorbate-2-phosphate (500 ng/ml (*v*/*v*)), and Na_2_HPO_4_/NaH_2_PO_4_ (3 mM (*v*/*v*), pH 7,4, Merck) as phosphate source. The histological fixation in 4% formalin solution occurred on days 28 and 42. The samples were stained 10 minutes in the dark with 0.5% Alizarin-Red S (Fluka, Germany) at pH 4.5. The quantitative analysis was performed in the same way as described for the adipogenesis (section 2.4.1).

#### 2.4.3. Chondrogenic *In Vitro* Differentiation

For chondrogenic differentiation, 250,000 hBMSCs were resuspended in control medium (DMEM FG0435 (500 ml (*v*/*v*), 4-(2-hydroxyethyl)-1-piperazineethanesulfonic acid (HEPES, 20 mM (*v*/*v*)), penicillin/streptomycin (1% (*v*/*v*)), dexamethasone (0,1 *μ*M (*v*/*v*)), Insulin-Transferrin-Selenium (ITS, 10 *μ*l/ml (*v*/*v*), Sigma Aldrich), ascorbate-2-phosphate (170 *μ*M (*v*/*v*), Sigma Aldrich), sodium-pyruvate (1 mM (*v*/*v*), Biochrom), and proline (350 *μ*M (*v*/*v*), Roth) and centrifuged to generate the required pellet form. Afterwards, the pellets were cultivated in differentiation medium (control medium with additional transforming growth factor-*β*3 (TGF-*β*3, 10 ng/ml/1.1 *μ*l (*v*/*v*), Peprotech)). Media were replaced every 7 days. For histological analysis, differentiation was stopped on days 7, 14, 21, and 28, and were pellets fixed in 4% formalin solution, embedded in Tissue Tek (OTC blue, Sakura Finetek, Staufen, Germany), and frozen in liquid nitrogen. Histological slices of 5 *μ*m were generated with a cryomicrotome (Microm HM 500 OM, Microm, Walldorf, Germany) and transferred onto coated microscope slides (SuperFrost Plus, Thermo Scientific, Darmstadt, Germany). After desiccation, samples were dyed with 0.1% safranin-O solution (Merck) for 15 minutes. After digitalization, the quantitative analysis was performed using two different groups to determine the successful progress of chondrogenesis. Chondrogenesis was graded as positive or negative differentiation. For evaluation, the different zones in the samples were marked. Afterwards, a grid was laid over the picture, and the boxes in the area of the two different groups were counted with a manual cell counter. As a result, the percentage of each group per chondrogenesis pellet was generated.

## 3. Statistical Analyses

Statistical analyses were performed using SPSS Statistics®, version 24 (IBM SPSS Statistics Corp., New York, NY). All data were nonparametric. The primary outcome of the study was to detect significant differences between the three different storage conditions. Therefore, comparisons between the groups were performed using the Kruskal-Wallis test. Results are expressed as median and 95% confidence interval (e.g., 5% (CI: 3.90-9.53%)). Graphical results are shown as boxplots or dot plots. A *p* value of ≤ 0.05 was considered significant.

## 4. Results

### 4.1. Cell Amount and CFU-F Assay

**Figure 1:**
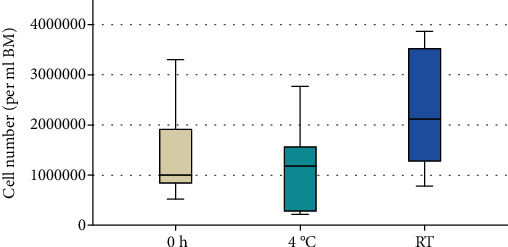
Cell number per ml BM in P0. The total number of cells per ml BM in P0 was determined. There was no significant difference between the groups (*p* = 0.095; *n* = 6).

The total number of cells per ml bone marrow (BM) was calculated after immediate and delayed processing of the BM (Figure 1). No significant differences were detected between the groups (*p* = 0.095).

**Figure 2:**
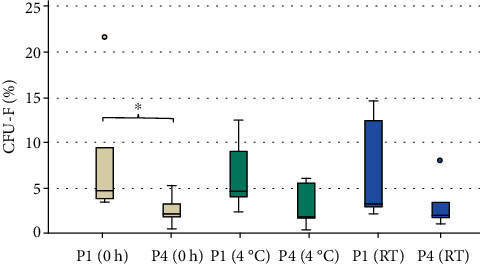
Percentage of CFU-F of the three experimental groups in P1 and P4. A significant difference was detected in group 0 h comparing passage 1 and 4 (^∗^*p* = 0.015; *n* = 6).

The self-renewal capacity of hBMSCs was evaluated with CFU-F assays in P1 and P4 (Figure 2), and no significant differences were seen between the three different isolation groups. After immediate isolation of the BMSC (0 h), 5% (CI: 3.90-9.53%) of all seeded cells formed a CFU. This is a significant difference compared to P4 of the same group in which only 2% (CI: 1.90-3.30%) of the cells were able to form a colony (*p* = 0.015). No significant differences were detected between P1 and P4 for groups 4°C (P1: 5% (CI: 4.10-9.07%) and P4: 2% (CI: 1.77-5.67%; *p* = 0.132)) and RT (P1: 3% (CI: 2.90-12.40%) and P4: 2% (CI: 1.77-3.50%; *p* = 0.177)).

### 4.2. *In Vitro* Differentiation of hBMSCs

#### 4.2.1. Adipogenic Differentiation

**Figure 3:**
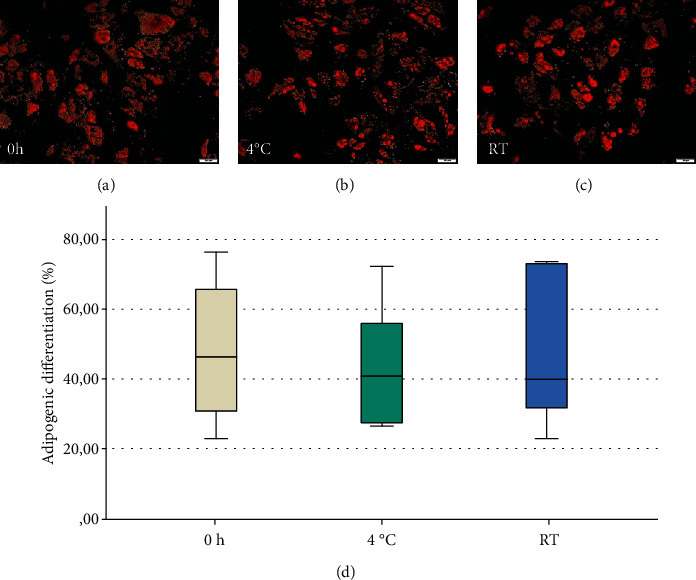
Adipogenic differentiation after 28 days. (a–c) Positive adipogenic differentiation was detected in all three groups ((a): 0 h; (b): 4°C; and (c): RT) after staining with Oil Red O. The scale bar corresponds to 50 *μ*m. (d) No significant difference regarding the adipogenic differentiation potential was detected (*p* = 0.796; *n* = 6).

All stem cells which were cultured in the adipogenic differentiation medium exhibited an adipogenic differentiation (Figures 3(a)–3(c)). All control groups were negative. On day 28, the median of the three groups showed almost the same value with 46% (CI: 31–66%) in group 0 h; 41% (CI: 27–56%) in group 4°C and 40% (CI: 32–73%) in group RT (*p* = 0.796; Figure 3(d)).

#### 4.2.2. Osteogenic Differentiation

**Figure 4:**
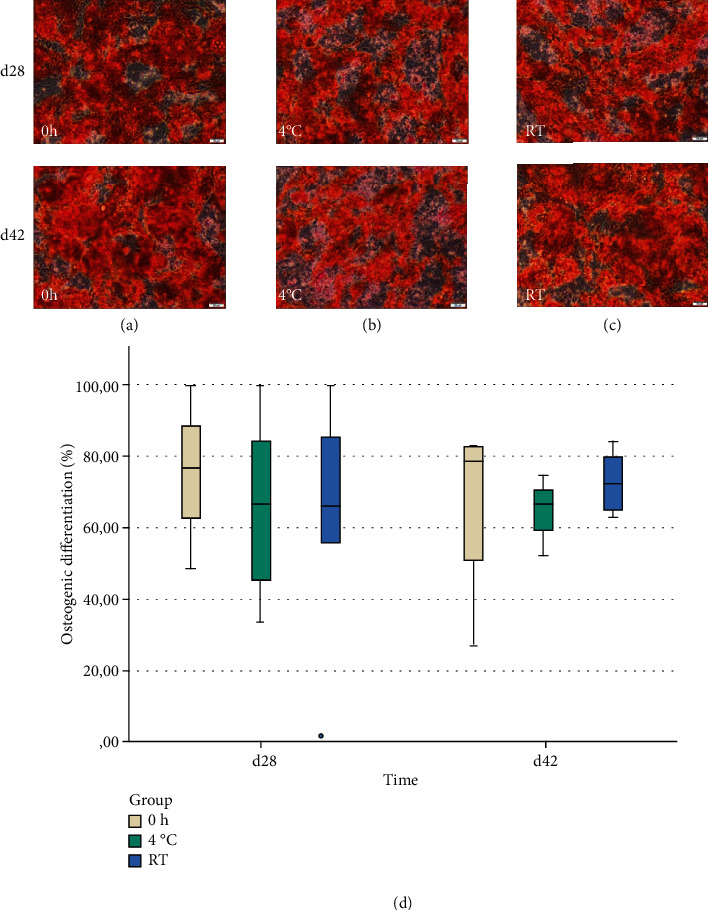
Osteogenic differentiation after 28 and 42 days after staining with Alizarin-Red S. (a–c) Osteogenesis was observed in all samples ((a): 0 h; (b): 4°C; and (c): RT) on days 28 (i) and 42 (ii). The scale bar corresponds to 50 *μ*m. (d) No significant differences between the groups were found on both examination dates (day 28: *p* = 0.803; day 42: *p* = 0.472; *n* = 4).

All stem cells which were cultured in the osteogenic differentiation medium exhibited an osteogenic differentiation on days 28 and 42 (Figures 4(a)–4(c)). All control groups were negative. For both time points, no significant differences between the storage conditions (day 28: *p* = 0.803 and day 42: *p* = 0.472) were detected as shown in Figure 4(d).

#### 4.2.3. Chondrogenic Differentiation

**Figure 5:**
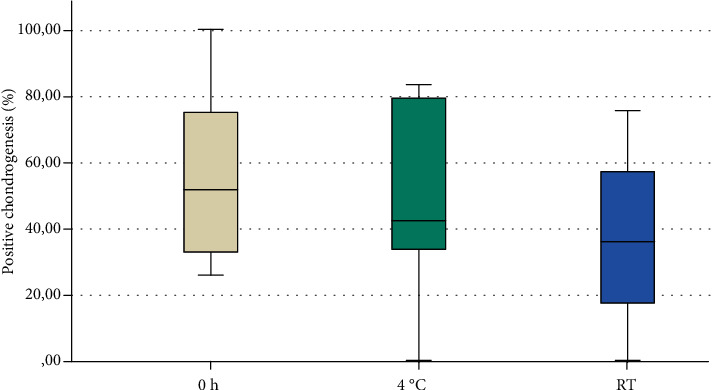
Positive chondrogenic differentiation after 28 days. The statistical examination revealed no significant difference regarding positive chondrogenesis (*p* = 0.590).

Chondrogenesis was observed in all induced groups except in two samples of different donors (one at room temperature and one at 4°C). All control groups were negative. The examination of the positive chondrogenesis revealed no significant differences between the three groups (0 h: 51.62% (CI: 33–75%); 4°C: 42.55% (CI: 34–79%); RT: 36.32% (CI: 18–57%); *p* = 0.590; Figure 5).

### 4.3. Flow Cytometry (FC)

The expression of 13 different surface proteins were analysed by flow cytometry. Regarding the expression of CD13, CD29, CD44, CD73, CD90, CD105, and CD166, the percentage median was over 90%, and the surface proteins CD11b, CD15, CD31, CD34, and CD45 were expressed less than 10% in median on the stem cells (Table 2).

The Kruskal-Wallis test demonstrated no significant differences in expression of the reviewed positive (CD13: *p* = 0.241; CD29: *p* = 0.821; CD44: *p* = 0.566; CD73: *p* = 0.873; CD90: *p* = 0.738; CD105: *p* = 0.587; and CD166: *p* = 0.474) and negative (CD11b: *p* = 0.800; CD15: *p* = 0.675; CD31: *p* = 0.531; CD34: *p* = 0.907; and CD45: *p* = 0.920) surface proteins between the three experimental groups.

## 5. Discussion

The seeding of MSCs onto a scaffold to treat large bone defects is an important research area in orthopeadics, but in the clinical setting, it is not always possible to immediately isolate stem cells out of the bone marrow for research purposes.

Therefore, the aim of this study was to investigate the influence of an overnight storage of bone marrow at different temperatures on the characteristics of hBMSCs. To the best of our knowledge, there are no current publications that describe various storage conditions for bone marrow aspirates prior to isolation of the hBMSC. However, we have found some publication on the effects of various storage conditions after the isolation of the hBMSCs.

Sohn et al. investigated the influence of the duration of *in vitro* storage from MSCs on viability, self-renewal, and differentiation capability at 4°C and room temperature. With a longer time period, the differentiation potential was decreasing. The storage temperature had no significant influence regarding the cell quality [18]. This fact is congruent to our results where no significant difference was found comparing storage at 4°C and room temperature.

Among other parameters, another study compared the influence of different temperatures (4°C, 37°C, and room temperature) on viability, differentiation capability, and expression of surface antigens of freshly and frozen-thawed MSCs. Storage of MSCs at room temperature or 37°C showed a lower viability than 4°C. The analysis of surface antigens showed a similar expression pattern for fresh MSCs independent of the storage condition (4°C or room temperature) [19].

According to the minimal criteria for defining MSCs from the ISCT, the defining positive markers (CD73, CD90, and CD105) were also expressed on the MSCs in all groups in our attempt [20]. In addition to these positive markers, we examined surface proteins which should be negative on MSCs. The negative expression was congruent to results of other publications [17, 20, 21]. The expression of HLA-DR was not measured because the addition of FGF2 to MSC growth medium is known to induce the expression of this surface antigen [22].

To evaluate the self-renewal of the isolated hBMSCs, the CFU-F assay was used which is a scientifically established method to measure the clonogenic potential in a quantitative form [23, 24]. The comparison of the three experimental groups within passages 1 and 4 revealed no statistical significant differences. Thus, we assumed that the examined storage conditions had no influence on the self-renewal of the stem cells. In passage 1, a higher number of colonies was observed than in passage 4 in all groups with statistical significance for group 0 h. This circumstance is due to the replicative senescence of cells in culture which was also described by Schellenberg et al. In their experiment, the frequency of colony forming units declined with increasing number of cell passages [25].

At least some other previous studies examined the possibility of stem cell isolation from other tissue sources after storage. Perry et al. showed that the isolation of dental pulp-derived MSCs is possible for five days after tooth extraction and storing at 4°C and implied that an immediately preparation is not mandatory [26]. Adipose-derived stem cells can be used for long-term storage after isolation from adipose tissue. The sample should be stored at room temperature and used within 24 hours [27].

## 6. Conclusion

In summary, the results assumed no distinct favorite role of one examined storage condition for bone marrow aspirate. This indicates that a storage of bone marrow aspirates for 18 to 24 hours at room temperature or 4°C is possible without loss of stem cell characteristics concerning the adipogenic, chondrogenic, and osteogenic differentiation, as well as the self-renewal capacity and the expression of the evaluated surface proteins.

## Figures and Tables

**Table 1:** Fluorochrome-conjugated antibodies against CD antigens for flow cytometry analysis.

Antigen	Dye	Ig-class	Clone	Host	Company
CD11b	APC	IgG1 k	ICRF44	Mouse	Biolegend
CD13	APC-Cy7	IgG1 k	WM15	Mouse	Biolegend
CD15	FITC	IgG1 k	W6D3	Mouse	Biolegend
CD29	APC	IgG1 k	TS2/16	Mouse	Biolegend
CD31	FITC	IgG1 k	WM59	Mouse	Biolegend
CD34	PE-Cy7	IgG1 k	581	Mouse	Biolegend
CD44	FITC	IgG1 k	BJ18	Mouse	Biolegend
CD45	APC-Cy7	IgG1 k	H130	Mouse	Biolegend
CD54	PE	IgG1 k	HCD54	Mouse	Biolegend
CD56	PE-Cy7	IgG2a k	MEM-188	Mouse	Biolegend
CD73	APC	IgG1 k	AD2	Mouse	Biolegend
CD90	PerCP Cy5.5	IgG1 k	5E 07	Mouse	Biolegend
CD105	PE	IgG1 k	43A3	Mouse	Biolegend
CD106	APC	IgG1 k	STA	Mouse	Biolegend
CD146	PE-Cy7	IgG2a k	SHM-57	Mouse	Biolegend
CD166	FITC	IgG1 k	3A6	Mouse	MBL
CD271	PerCP Cy5.5	IgG1 k	ME20.4	Mouse	Biolegend
CD274	PE	IgG2b k	29E.2A3	Mouse	Biolegend
HLA-DR	PE	IgG2b k	LN3	Mouse	Biolegend
Stro-1	APC	IgM k	STRO-1	Mouse	Biolegend

**Table 2:** Expression of specific surface proteins. Positive expression with a median higher than 90% was observed for the surface proteins CD13, CD29, CD44, CD73, CD90, CD105, and CD166 in all groups. The surface proteins CD11b, CD15, CD31, CD34, and CD45 were expressed less than 10% in median on stem cells.

Surface protein	0 h			4°C			RT		
	Median	*Q*_1_	*Q*_3_	Median	*Q*_1_	*Q*_3_	Median	*Q*_1_	*Q*_3_
CD13	97.4	95.0	98.0	98.1	97.0	98.4	98.0	97.1	98.4
CD29	98.6	97.3	100.0	98.5	97.0	99.0	98.7	98.0	99.0
CD44	98.6	97.8	100.0	98.2	97.9	98.8	98.3	97.3	98.3
CD73	98.8	98.3	99.6	98.5	98.2	99.6	98.7	98.3	98.8
CD90	95.9	94.7	99.0	96.7	88.1	97.6	95.3	87.1	97.6
CD105	99.3	98.4	100.0	98.6	98.4	99.1	98.8	98.7	99.0
CD166	98.5	98.0	99.0	98.7	98.6	99.0	98.6	98.6	98.6
CD11b	1.1	0.0	1.6	1.0	1.0	2.0	1.4	0.4	2.0
CD15	0.4	0.2	1.0	0.7	0.2	1.4	0.4	0.0	1.0
CD31	1.8	1.2	2.6	3.1	1.4	4.6	2.7	1.6	5.7
CD34	0.8	0.0	5.0	1.0	0.0	7.0	1.1	0.0	7.0
CD45	1.1	0.4	3.0	1.5	0.5	1.8	1.1	0.9	2.0

## Data Availability

The datasets generated during and/or analysed during the current study are available from the corresponding author on reasonable request.
